# Highlight: The Colorful History of Plastids

**DOI:** 10.1093/gbe/evaa116

**Published:** 2020-07-10

**Authors:** Casey McGrath

A billion years ago, a single-celled eukaryote engulfed a cyanobacterium—an organism capable of converting the sun’s energy into food in the form of carbohydrates. In one of the single most pivotal events in the history of life, instead of the bacterium being digested, an endosymbiosis was formed, with the bacterial cell persisting inside the host eukaryote for millennia and giving rise to the first photosynthetic eukaryotes. The descendants of this merger include plants, as well as a large number of single-celled eukaryotes that are collectively referred to as algae (i.e., kelp, nori). The remnants of the cyanobacterium eventually evolved into an organelle known as a plastid or chloroplast, which allows photosynthetic eukaryotes to produce their own food—and thus to provide food to animals like us. Despite the importance of this event, a variety of aspects of plastid evolution have long remained shrouded in mystery. In a review in *Genome Biology and Evolution*, Shannon Sibbald and John Archibald highlight emerging genome data in this field and provide new insight into plastid evolution (Sibbald and Archibald 2020).

**Fig. 1.**
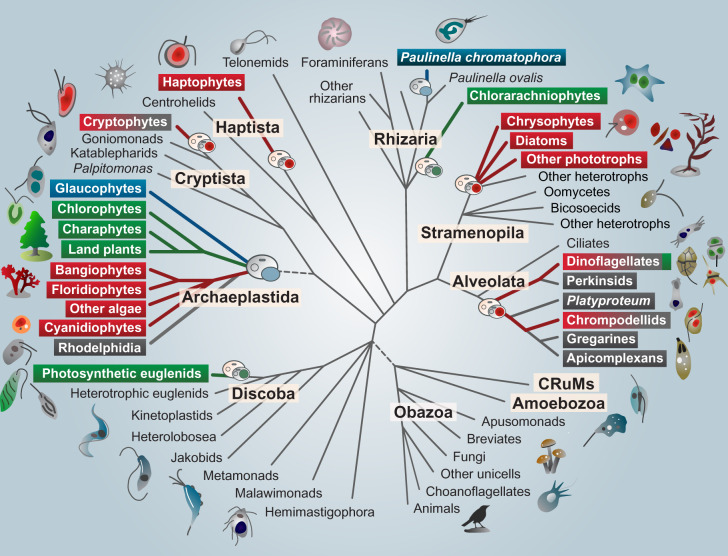
Schematic evolutionary tree of eukaryotes with an emphasis on photosynthetic lineages. Members of the Archaeplastida have primary plastids stemming directly from cyanobacteria. The primary plastids of red and green algae have spread from the Archaeplastida to other branches of the tree, including Rhizaria, Discoba, and Stramenopila. The colored taxon names in these lineages reflect the green or red algal secondary or tertiary endosymbiotic origin of their plastids. Taxon names highlighted in gray indicate the presence of one or more secondarily nonphotosynthetic members.

Plastids can be found patchily distributed across a number of eukaryotic lineages (fig. 1), often nested within groups that are otherwise nonphotosynthetic. This irregular distribution has historically made it difficult for scientists to uncover the evolutionary origin and history of plastids and to trace their transfer from branch to branch on the eukaryotic tree. Both biochemical and molecular data point to a single origin of plastids from the engulfment of a member of the β-cyanobacteria by the single-celled ancestor of the Archaeplastida, a group consisting of all green algae (including land plants), red algae, and glaucophyte algae.

Secondary, tertiary, and higher order acquisitions of plastids are thought to have occurred when a eukaryotic cell containing a plastid was then engulfed by another eukaryote, which was then engulfed by yet another eukaryote, and so on. This type of plastid acquisition appears to have involved green algal endosymbionts on at least two occasions and perhaps even more often with red algae, giving rise to so-called “complex” plastids. According to Archibald, “Multigene phylogenies have gradually improved our understanding of the structure of the eukaryotic tree, and, consequently, the field has started to shift—the emerging consensus is that secondary/tertiary endosymbiosis is more common than previously assumed.” However, coauthor Sibbald points out that this does not mean that the puzzle has been solved. “We still don’t know how many secondary, tertiary, and higher endosymbiotic events gave rise to the plastid diversity we see today or, in many cases, who the host and endosymbiont partners were.”

Even more confusing, recent data show that the majority of eukaryotes with plastids acquired from red or green algae (i.e., complex red or green plastids) actually harbor a mosaic of genetic material from both red and green algae in their nuclear genomes. This has led to the “shopping bag” model of plastid evolution, which suggests that multiple red and green algae may have been transiently housed by various eukaryotic lineages over time before the establishment of the permanent (or at least current) resident plastid. Indeed, a number of lineages participate in a practice known as “kleptoplasty,” in which they consume plastid-containing algae and steal their plastids, harboring them for several weeks to months and benefiting from the carbohydrates they produce before replenishing them with new ones. Thus, although it seems at least plausible that some lineages may have harbored a mix of both red and green algae before one eventually became more permanently established, Archibald notes that “understanding the evolutionary significance of this ‘red-green’ mosaicism in modern-day algae remains a particularly challenging task.”

Additional difficulties in tracing plastid evolution arise from the loss of photosynthesis in some lineages, which can be accompanied by the further loss of the plastid genome and, in a few cases, complete loss of the plastid itself. For example, most apicomplexans like the malaria parasite *Plasmodium* contain a relict plastid-like organelle known as the apicoplast, which still carries out a number of biosynthetic processes. In the closely related human parasite *Cryptosporidium parvum*, however, the plastid has been completely lost (or was never present), and key metabolites are instead scavenged from the host. There are also a number of plants that have lost the ability to photosynthesize and instead parasitize other plants or rely on fungi to provide nutrients, such as the parasitic flowering plant *Rafflesia*.

Perhaps most interestingly, the tenet that all plastids stem from a single eukaryotic-cyanobacterial endosymbiosis has even been called into question by recent data showing that the freshwater shell-building ameba *Paulinella chromatophora* possesses photosynthetic organelles derived from an independent acquisition of an α-cyanobacterium, which is thought to have occurred only a hundred million years ago. This suggests that more widespread sampling may reveal other lineages with independently derived plastid-like organelles. These more recent events may provide new insight into the mechanisms by which a cyanobacterium evolves into a plastid within the host cell.

Rather than answering the decades-old questions in the field of plastid evolution, it appears that recent genomic data have for now revealed even more unknowns. As Archibald notes, “It is sobering to consider that as we have collected more sequence data, the picture of plastid evolution has become less and less clear.” Answering these questions will require addressing what Archibald describes as “a longstanding challenge in the field”: integrating data from independent lines of inquiry, including phylogenetics, biochemistry, and membrane biology.
